# Temporal hierarchy of observed goal-directed actions

**DOI:** 10.1038/s41598-023-46917-z

**Published:** 2023-11-11

**Authors:** Shahar Aberbach-Goodman, Roy Mukamel

**Affiliations:** Sagol School of Neuroscience and School of Psychological Sciences, Tel Aviv University, 6997801 Tel-Aviv, Israel

**Keywords:** Motor control, Perception

## Abstract

During social interactions, we continuously integrate current and previous information over varying timescales to infer other people's action intentions. Motor cognition theories argue for a hierarchical organization of goal-directed actions based on temporal scales. Accordingly, transient motor primitives are represented at lower levels of the hierarchy, a combination of primitives building motor sequences at subordinate levels, and more stable overarching action goals at superordinate levels. A neural topography of hierarchal timescales for information accumulation was previously shown in the visual and auditory domains. However, whether such a temporal hierarchy can also account for observed goal-directed action representations in motor pathways remains to be determined. Thus, the current study examined the neural architecture underlying the processing of observed goal-directed actions using inter-subject correlation (ISC) of fMRI activity. Observers (n = 24) viewed sequential hand movements presented in their intact order or piecewise scrambled at three timescales pertaining to goal-directed action evolution (Primitives: ± 1.5 s, Sub-Goals: ± 4 s, and High-Goals: ± 10 s). The results revealed differential intrinsic temporal capacities for integrating goal-directed action information across brain areas engaged in action observation. Longer timescales (> ± 10 s) were found in the posterior parietal and dorsal premotor compared to the ventral premotor (± 4 s) and anterior parietal (± 1.5 s) cortex. Moreover, our results revealed a hemispheric bias with more extended timescales in the right MT+, primary somatosensory, and early visual cortices compared to their homotopic regions in the left hemisphere. Our findings corroborate a hierarchical neural mapping of observed actions based on temporal scales of goals and provide further support for a ubiquitous time-dependent neural organization of information processing across multiple modalities.

## Introduction

Humans perform voluntary actions to accomplish goals spanning varying complexities and temporal scales. Although complex, the goals of observed action sequences are effortlessly inferred by typically developing individuals during social interactions. Despite three decades of studies of the brain areas involved in the processing of observed actions, known as the action observation network (AON)^1,2^, there is currently no consensus in the literature regarding the organizing principle according to which observed sequences of goal-directed actions are parsed and mapped in the brain. Behaviorally, it was shown that people utilize the information of observed kinematics to infer other's intentions^3^. At the neural level, several brain regions within the AON have been implicated to play a role in predicting action goals from the observed kinematics, including the superior parietal lobule (SPL), inferior frontal gyrus (IFG), middle frontal gyrus (MFG), and the inferior parietal lobule (IPL), with the latter contributing the most to goal prediction^4^. Moreover, previous studies show that the feature of the realized goal or outcome of observed actions is also encoded in fronto-parietal regions, independent of the action's kinematic features^5–7^.

The neuroimaging findings of goal encoding are in line with behavioral findings from children, showing enhanced learning from imitation of new action sequences when provided with knowledge about the overarching goals that bind the actions together^8^. However, considering that observed actions are encoded in neural activity both at the kinematic as well as at the conceptual goal level^9^, it is yet unclear how the intention-specifying information conveyed by action kinematics is accumulated and integrated to encode the overarching action goals.

Previous neuroimaging studies have argued for a hierarchical neural organization of observed actions. Findings from a series of repetition suppression fMRI studies^7,10,11^ showed sensitivity for observed low-level kinematics of hand-object interactions in the inferior occipital cortex and the SPL, while the premotor cortex and the intraparietal sulcus (IPS) were shown to be sensitive to objects and action outcomes (e.g. open/closed box), regardless of the performed movement (e.g., pushing/pulling). The levels of action representation in the ventral premotor cortex (PMv) and IPS were later dissociated in additional fMRI studies of action observation, showing low-level, concrete, action representation in the PMv while action encoding was generalized across different perceptual-object and kinematic features in the IPS and the lateral occipital temporal cortex (LOTC)^12,13^. These findings indicate hierarchical levels of the neural representation of goal-directed actions within action observation pathways—a concrete representation of the movement's perceptual-sensory and motor features in motor/premotor areas, and an action goal representation that is generalized across these physical attributes in parietal cortices. Nevertheless, there is no consensus regarding the specific relation between the levels of the hierarchy (e.g., the kinematics, the manipulated object, the visual outcome, etc.) or the level at which each aspect is represented relative to the others (i.e., objects at the same or higher level than kinematics). Moreover, the actions examined in these studies consisted of a discrete outcome. Thus, the neural substrates for processing natural continuous action sequences, comprised of multiple sub-actions directed to an overarching goal, remain an open question.

Other than arguments for an action hierarchy based on the abstraction of sensory-motor features, Uithol and colleagues argued for a hierarchy of temporal stability for the organization of goal-directed action sequences that is governed by the actions' dynamics over *time*^14^. Specifically, that high levels of the hierarchy encode more stable (goal-related) representations, whereas lower levels represent more transient (motor primitives) kinematics. Accordingly, different brain regions are postulated to have different intrinsic time scales on which they can represent sequential contingencies.

Previous studies, using Inter-Subject Correlation (ISC)^15,16^ revealed a topographical mapping of processing time scales in brain areas of the auditory and visual systems^17–19^. Specifically, the authors defined a temporal receptive window (TRW) for a given region as the length of time during which sensory information is integrated and may affect the response in that region. In their findings, TRW sizes were shown to increase from primary to high-level perceptual and cognitive areas. Accordingly, the shortest TRWs (< ~ 4 s) for visual or auditory stimuli were found in the corresponding early sensory regions (V1/MT+, and A1), while long TRWs (> ~ 30 s) were found in the frontal eye field (FEF) and temporal-parietal junction (TPJ) for visual and auditory stimuli, respectively. These studies support a neural representation of hierarchical information accumulation according to temporal scales. Interestingly, areas engaged in action observation were shown to be sensitive to the predictability of action information over time^20,21^. Therefore, a potential principle linking the different hierarchical levels of goal-directed actions may be a temporal processing capacity. However, such a temporal organization of hierarchical observed (and executed) goal-directed actions is currently mainly supported by computational models and action recognition of human every-day activities^22–24^. Thus, a precise neural mapping of levels in the temporal hierarchy, encoding goal-directed actions, in sensory-motor pathways is still missing.

To this end, in the current study we adapted the ISC approach to examine whether brain regions previously found to encode action goal information have different intrinsic time scales for processing observed actions. Specifically, we manipulated the sequence coherency of observed goal-directed hand actions at three timescales: basic motor primitives, Sub-Goals, and High-Goals. Building on previous work indicating that areas involved in the processing of observed actions are responsive to different action elements, we hypothesized to find a graded neural topography of intrinsic processing time scales compatible with the representation of action goals across the areas sensitive to action observation. Action observation engages areas of the motor, somatosensory, and visual systems. Previous studies using similar methods suggested predictive coding as a potential mechanistic delineation of the feed-forward and feedback connections between different action observation areas^20,21^. These studies show that the PMv, IPS, and inferior parietal lobe (IPL) are sensitive to action predictability and therefore to the temporal coherency of action information. Nevertheless, it is yet unclear whether these frontoparietal regions have different temporal processing capacities and whether or not they are positioned at similar or different levels of the action hierarchy. We thus have no specific prediction regarding the topographical gradient direction of the TRW sizes. In addition to its sensitivity to action coherency, the parietal lobe is considered in the literature as an integrative hub for sensory-motor and visual information, and was indicated to have a prominent role in high-level action representation^25^ and coding of intention^26,27^. We thus hypothesized the regions of the parietal lobe to exhibit a relatively long TRW, allowing high-level goal representation that is more stable over time.

## Methods

### Participants

Twenty-eight subjects were recruited for the study. Three subjects were excluded from data analysis due to excessive movement in the scanner, and one due to low performance (see experimental procedure below). Accordingly, the data for the study was acquired from a final sample of 24 participants (15 females, mean age 24.8, range 18–34 years). All participants were healthy with no history of neurological or psychiatric disorders, right-handed (self-report), had normal or corrected-to-normal vision, and were naive to the purposes of the study. All methods in the study were carried out in accordance with the approved guidelines of the ethical committee at Tel-Aviv University and the Helsinki Committee of the Sheba Medical Center. All participants provided written informed consent to participate and were compensated for their time.

### Stimuli

**Figure 1:**
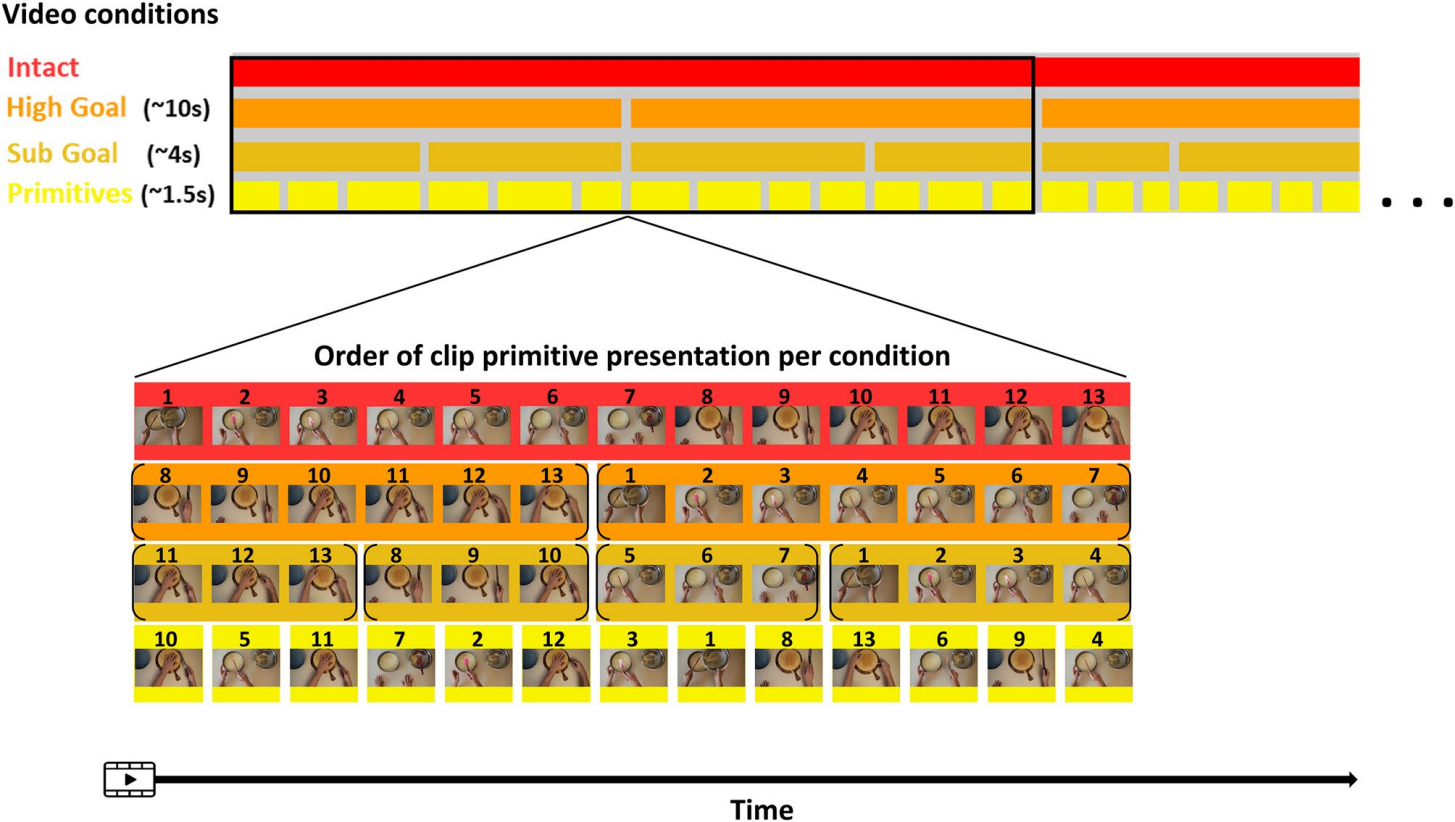
Task design. Participants observed four movies of goal-directed actions, performed by an actor preparing a cake. One video consisted of the actions in their intact order and the three other videos consisted of the actions scrambled at High-Goal (coherent information within each ± 10 s time bins of the movie), Sub-Goal (coherent information within each ± 4 s time bins of the movie), and Primitives (coherent information within each ± 1.5 s portion of the movie) time scales. In the illustration, each image represents a primitive movie clip with its number representing the clip temporal order. All four movies had a duration of 5 min and consisted of the same 200 primitive clips (concatenated in different ordering depending on the scramble condition).

The experimental stimuli consisted of four videos depicting the hand movements of a right-handed actor preparing a cake. The videos were edited using ADOBE Premiere Pro CC 15.0 and DaVinci Resolve 16. First, we constructed the *intact* video. This movie contained the actor’s movements in a chronological sequential order. Then, the intact movie was subdivided into clips containing basic motor primitives (e.g., grasping; mean duration 1.5 s ± 0.06 s). These clips were randomly reordered to construct the video for the shortest timescale scramble—the *Primitives* condition. For the two other timescale scramble conditions, several clips consisting of primitives such as *'reaching'*, *'grasping*', *'transporting the cup*', etc. were joined in their original chronological order to construct meaningful action goals at the *Sub-Goal* level (e.g. ‘*pouring the egg*'; mean duration 4.1 s ± 0.2 s), and *High-Goal* level (e.g. *'adding eggs to cake batter*'; mean duration 10 s ± 0.56) consisting of several sub-goals such as *'pouring the egg*', *'blending with batter’',* etc.). See Fig. 1 for illustration of study design and Table S1 for a complete list of primitives and action goals. Each of the four videos contained all primitive clips (with a different ordering depending on the condition's timescale) and had a total duration of 309 s. Representative 30 s snippets from each of the four experimental video conditions are provided in the supplementary materials S1–S4.

### Experimental procedure

Participants were presented with the four goal-directed action videos during four separate runs within a single fMRI session and were instructed to attend the videos. Each video was presented only once during a single scanning run. Video order was randomized and counterbalanced across participants. To assess participants' level of attention, following each video presentation they were requested to verbally answer a different yes/no question (e.g., *‘Did the actor drop something?’, ‘Did the actor rotate the cake?’, ‘Did the actor use scissors?’, ‘Did the actor clean the surface*?’). The data from one subject who failed to answer the question correctly on more than 2 videos was discarded from further analysis. The anatomical scan (MP2RAGE) was performed following the first two experimental runs. In each scan, a fixation mark was presented at the beginning and end of the video presentation. In order to prevent any predictability differences between the conditions with regard to the main goal of the actor in the videos, participants were informed at the beginning of the session that they will observe videos of a person preparing a cake. Videos were presented using Psychtoolbox-3 (Brainard 1997, www.psychtoolbox.org) on MATLAB 2016b (The MathWorks, Inc., Natick, MA, USA). Stimuli were presented on a 32′ monitor and viewed by the participants through a mirror placed on the MRI head coil.

### Image acquisition

Functional imaging was acquired on a Siemens Magnetom Prisma 3T Scanner (Siemens Healthcare) with a 64-channel head coil at the Tel-Aviv University Strauss Center for Computational Neuroimaging. An interleaved multiband gradient-echo, echo-planar pulse sequence was used in all functional scans. Whole-brain coverage was provided by acquiring 66 slices for each volume (slice thickness 2 mm; voxel size 2 mm isotropic; time to repetition (TR) = 1000 ms; time to echo (TE) = 30 ms; flip angle = 82°; field of view = 192 mm; acceleration factor = 2). For anatomical reference, a whole-brain high-resolution T1-weighted scan (slice thickness 1 mm; voxel size 1 mm isotropic; TR = 2530 ms; TE = 2.99 ms; flip angle = 7°; field of view = 224 mm) was acquired for each participant.

### Image preprocessing

fMRI data preprocessing was conducted using the FMRIB’s Software Library’s (FSL v5.0.7) fMRI Expert Analysis Tool (FEAT v6.00)^28^. The data from each experimental run underwent the following preprocessing procedures: brain extraction, slice-time correction, high-pass filtering at 100 s (0.01 Hz), motion-correction to the middle time-point of each run, smoothing with a 5-mm FWHM kernel, and correction for autocorrelation using pre-whitening (as implemented in FSL). Participants with an absolute displacement value exceeding 2 mm were excluded from analysis. All images were registered to the high-resolution anatomical data using boundary-based reconstruction and normalized to the Montreal Neurological Institute (MNI) template using nonlinear registration.

### Data analysis

#### Inter-subject correlation analysis

Inter-Subject Correlation (ISC) is a measure of reliability of the stimulus-locked neural response, in which the shared variance across subjects is used as a data-driven estimate of the evoked response. Accordingly, the time course of a voxel, or brain region, in a target subject is modeled with the time course of the corresponding location in a different source subject^15^. Brain activity in a single voxel or region of interest (ROI) can include three main components: a stimulus-evoked component that is common across subjects, a subject-specific stimulus-evoked component, and a stimulus-unrelated or noise component. By modeling one subject with another subject’s time course, the subject-specific and unrelated noise components are effectively filtered out—thus isolating the stimulus evoked responses that are shared across subjects^16^. Under the ISC approach, the level of engagement of a certain brain region during a task is determined according to the level of neural synchronization within that region across multiple subjects. The main advantage is that it allows the use of continuous dynamic naturalistic stimuli to discover the functional characteristics of different brain regions without requiring a pre-defined hemodynamic response model. The underlying logic of the ISC method is that, if a brain region is responsive to a certain stimulus and has the capacity to integrate over the continuous stimulus information, then randomizing the coherent order of the stimulus and destroying the high-level information reduces the processing efficacy in that region, and therefore decreases the degree of similarity (synchronization) across participants^20^.

#### Reliable voxels

To identify voxels with significant shared responses to the presentation of observed actions, we followed the leave-one-out approach for ISC analysis^16,19^. In this procedure, for each of the experimental videos, the time course of a given voxel in one subject was correlated with the average time course of the same voxel across all *remaining n-1* subjects (excluding that subject). The time course included all fMRI blood oxygenation level dependent (BOLD) volumes starting five seconds following movie onset until five seconds following movie offset (to account for the inherent delay of the hemodynamic response function)^17,29^. The analysis produced a whole-brain map of Pearson's linear correlation coefficients for each subject (in MNI space). Finally, a whole-brain group level ISC map was computed by averaging all individual maps, resulting in an average *r* value for each voxel. Only voxels with positive ISC values were considered in this analysis. The statistical significance of the group-level ISC values was computed using a phase randomization bootstrapping procedure^19^. Accordingly, we applied the Fourier transform to the empirical time series of each voxel (in each subject) and randomized the phase of the Fourier components. Then, we performed the inverse Fourier transformation, thus preserving the power spectrum of the signal but disrupting the temporal alignment^16^. We then computed a group ISC map in a similar manner to the way it was computed for the original data. This was repeated 1000 times, generating a null distribution of the correlation values. For each experimental video, the original ISC values were compared with their null distributions to compute the ISC *p* values. Finally, the *p* values across all experimental videos were corrected for multiple comparisons using the Benjamini–Hochberg false-discovery rate procedure (FDR)^30^, with *q* = 0.01. For the main comparison of ISC values across conditions, we used a mask that included all voxels that had significant ISC values in at least one of the experimental conditions.

#### ROI-based ISC and Paired-sample comparisons

To examine the temporal capacity of different cortical areas to accumulate information from observed, goal-directed actions, we parceled the brain into ROIs based on the Human Brainnetome Atlas^31^. Only ROIs that contained at least 30 voxels that reliably responded to at least one of the experimental videos (see voxel-based ISC analysis described above), were taken for further analysis. For each ROI included in this analysis, we computed the average time course across all significant voxels of each subject, separately for each of the four video presentations (i.e., Intact, High-Goal, Sub-Goal and Primitives). The ROI-based BOLD time courses were taken to compute the ISC in each condition using the same leave-one out approach described above for the analysis at the single voxel level.

**Figure 2:**
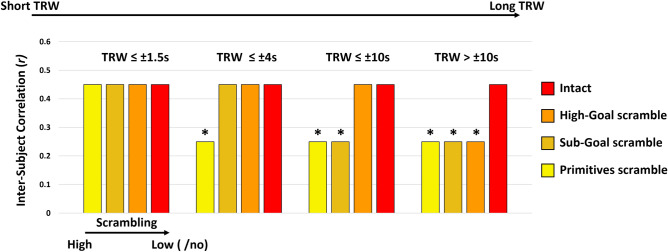
Schematic of four ISC patterns across conditions and their corresponding TRW types. The TRW of an ROI is determined according to the shortest time scale of scrambled videos (i.e., High-Goal/Sub-Goal/Primitives) for which the ISC was not significantly lower compared to the ISC in the intact (unscrambled) condition. *Significant reduction in ISC in scrambled compared to the intact condition.

For a given ROI, ISC should be highest for stimuli presented at a scrambling level that corresponds with the region's TRW. Presentation of scrambled stimuli at shorter time-scales than the optimal TRW will disrupt processing, and yield lower ISC values^16^. Presentation of scrambled stimuli at longer time-scales is not expected to disrupt processing, therefor ISC levels for longer time-scales should be similar (or at least not higher) than those of the optimal time-scale. The intact movie contains coherent information within all temporal time windows (i.e., short, intermediate and long time-windows). Therefore, the ISC value of the intact condition should be either maximal (in case the true TRW of the region is the longest time-scale examined), or not higher than the maximal ISC value obtained for a scramble condition (in case the true TRW of the region corresponds with a shorter time-scale). In order to identify the relative TRW type of each ROI at the group level, the ROI's ISC in the intact condition was compared to the ISC in each of the three scramble conditions using a one-tailed paired t-tests (ISC_Intact > ISC_scramble). Since correlation values do not follow a normal distribution, we performed a Fisher-z transformation of the ISC values prior to conducting the paired-sample t-tests. The TRW of each ROI was then determined according to the shortest time scale of scrambled videos, for which the ISC was not significantly smaller than the ISC in the intact (unscrambled) condition. For example, if a certain region does not show lower ISC levels in any of the scrambled conditions compared to the Intact, this region would be considered to have the shortest TRW—corresponding to the representation of the level of motor Primitives alone (≤ ~ 1.5 s). Similarly, a region exhibiting a reduction in ISC compared to the intact video, only for the Primitives condition but not for the higher time scales of Sub-Goal and High-Goal scrambles, would be considered to have a higher TRW—representing the observed information at the Sub-Goal and primitives level (≤ ~ 4 s). According to this rational, a region with a certain TRW will be considered sensitive to the information presented within the time scale of that TRW and all time scales beneath it, i.e., a region with a long TRW representing high-goals, also represents the lower-level sub-goal and primitives, that unfold within shorter time windows. See Fig. 2 for a schematic depiction of different ISC patterns and their corresponding TRW levels.

## Results

### Voxel-based ISC analysis (whole brain)

**Figure 3:**
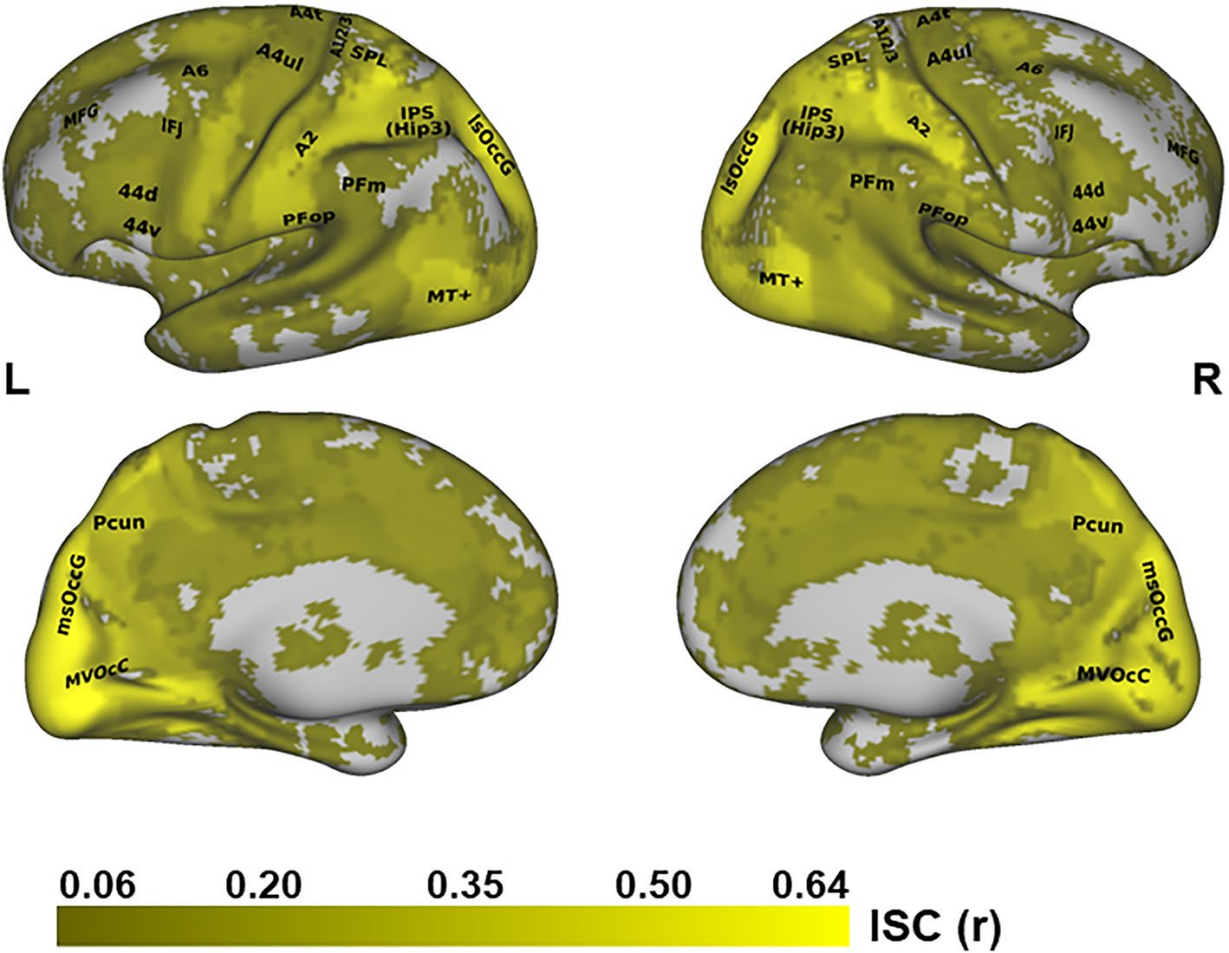
Voxels reliably responsive across subjects during action observation. Lateral (top) and medial (bottom) maps of ISC values in significantly responsive voxels during at least one of the experimental video presentations (tested versus a shuffle distribution of ISC values). *L* left hemisphere, *R* right hemisphere. Regions names on map according to the Brainnetome Atlas nomenclature.

In order to examine the sensitivity of different regions to the temporal scales of observed goal-directed actions, we first identified the voxels that reliably responded to observed actions during *at least one* of the videos (see “Methods”). The voxel based ISC analysis of the BOLD response revealed voxels that are significantly correlated across participants (p < 0.01, FDR corrected) in extensive portions of the brain, including cortical and sub-cortical regions. ISC values were highest in early visual cortices, MT+, lateral superior occipital gyrus, superior parietal lobule and the somatosensory cortex, see Fig. 3.

### ROI based ISC analysis

In order to identify the temporal capacity of different brain regions to accumulate information during observation of goal-directed actions, we extended our analysis from the single voxel level to the level of ROI. To this end, in each ROI for each subject, we first averaged the time series of the reliable voxels identified from the voxel based ISC analysis described above. Next, we computed the ISC value in each condition using the average ROI time course. Only ROIs with more than 30 reliable voxels were analyzed (see Table S2 for the complete list of ROIs and their ISC values in the four video conditions). In the current analysis, areas in which neural activity is primarily driven by transient dynamics of the stimuli (i.e., short TRW), would not exhibit a difference in ISC across the scrambled video conditions compared to the Intact video presentation. In contrast, in brain regions where activity depends on information accumulated over several seconds, the ISC should decrease with increasing scrambling levels (see “Methods” for the full schematic explanation).

### ISC values across different brain regions and timescales of observed goal-directed actions

#### Long TRW

**Figure 4:**
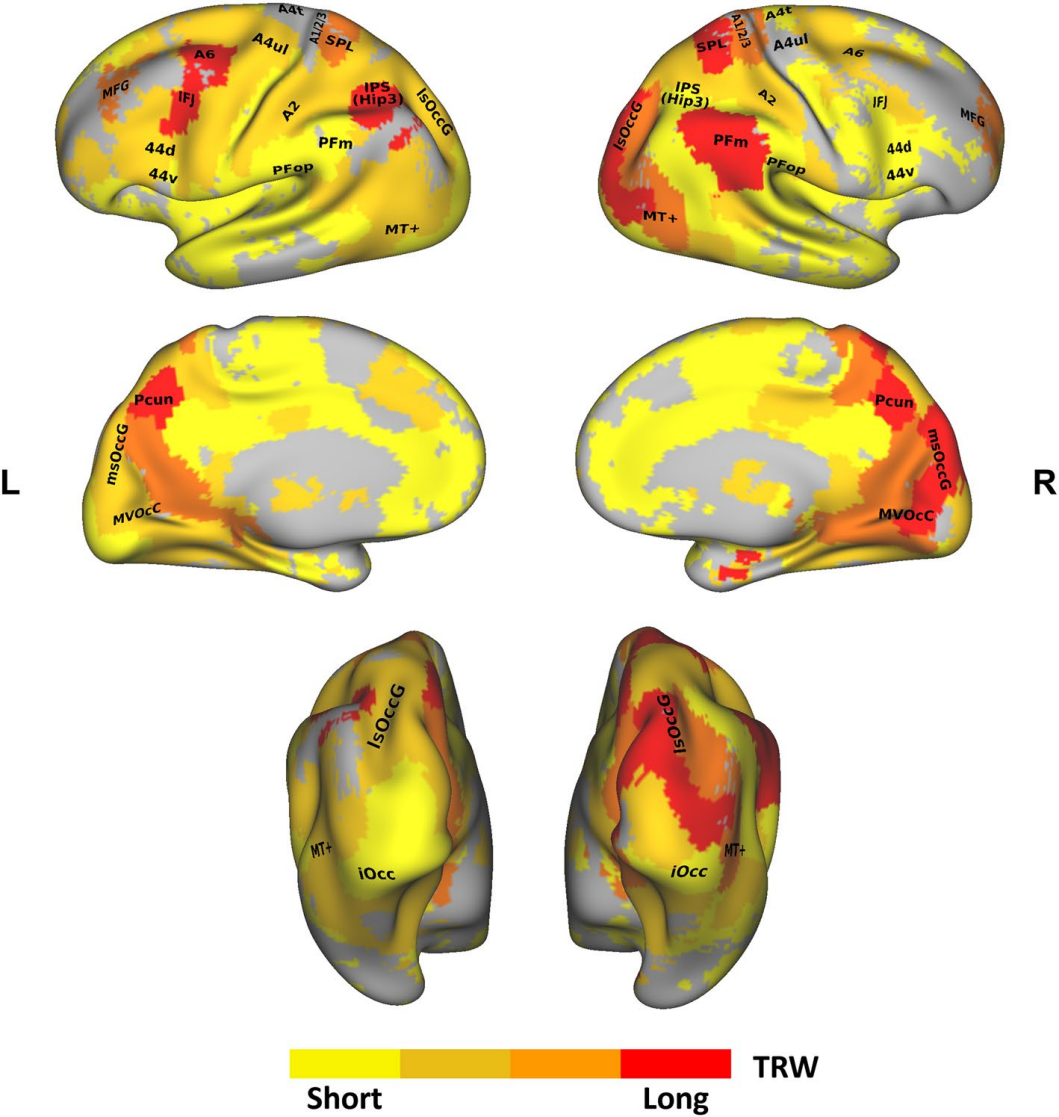
Hierarchical topography of TRWs across ROIs*.* Each ROI in the maps is colored according to its TRW type, as revealed by the paired sample comparison results, and determined according to the shortest time scale of scrambled videos in which the ISC was not lower compared to the intact (unscrambled) condition. *L* left, *R* right. Regions names on map according to the Brainnetome Atlas nomenclature (see “Methods”).

**Figure 5:**
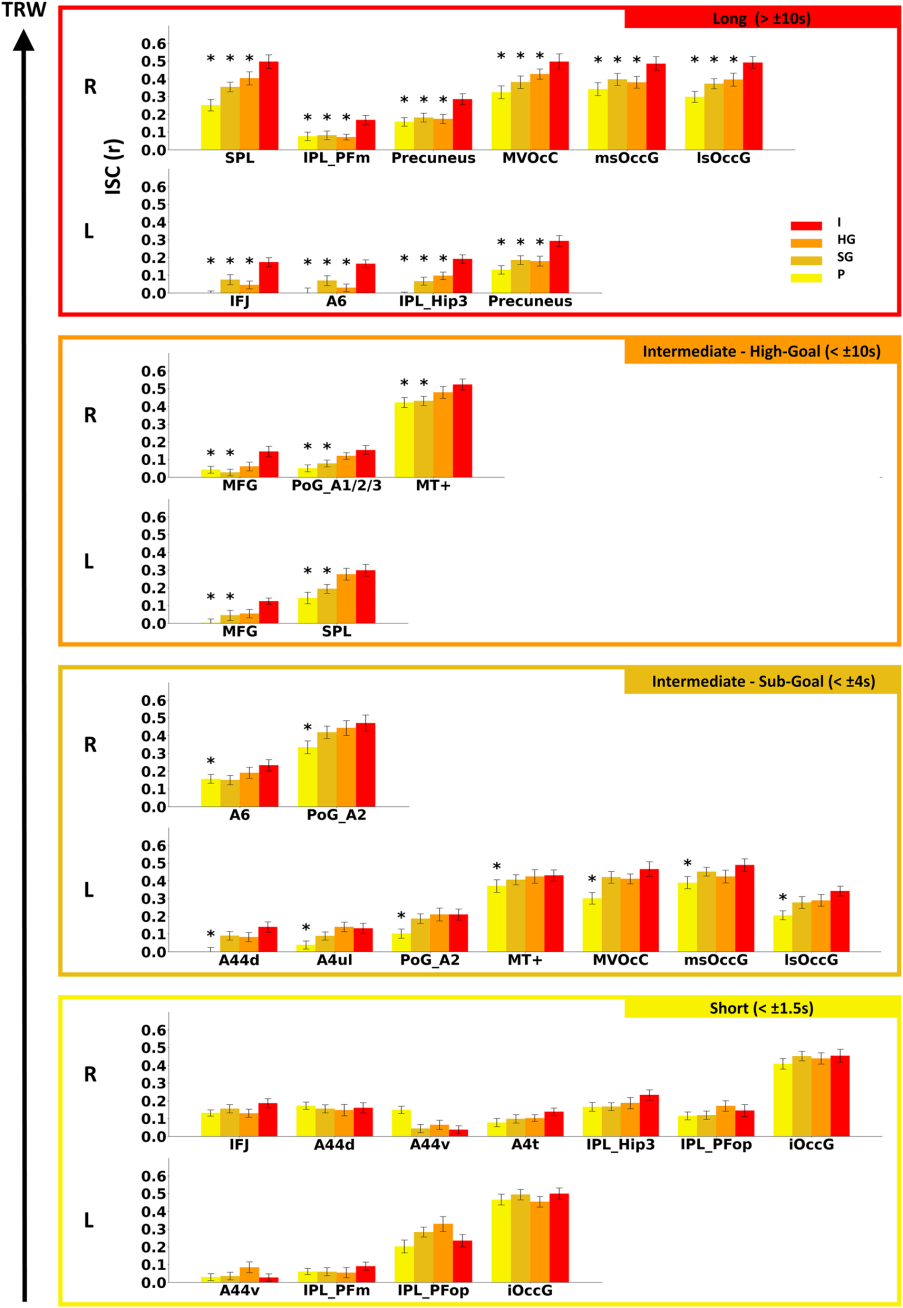
TRW hierarchy according to ISC values in several of the ROIS. ISC values in each movie condition for the right and left hemispheres (upper/lower row in each panel respectively). Regions with the longest to shortest TRWs are presented from top to bottom panels. Note the differences in absolute ISC levels across regions. Importantly, the TRW is determined by the relative pattern of correlation across conditions within the same region. *P* primitives, *SG* sub-goal, *HG* high-goal, *I* intact.

The longest TRWs, of more than ± 10 s, were found bilaterally in the Precuneus (A7), in the left inferior frontal junction (IFJ) and left dorsal premotor (PMd) cortex (A6) of the middle frontal gyrus (MFG) and in the left IPS (Hip3). In the right hemisphere, the longest TRWs were also found in the SPL and PFm of the posterior inferior parietal lobule. In these areas, ISC values were significantly lower in all scrambled conditions compared to the intact condition. In addition, the right lateral superior occipital gyrus (lsOccG), and early visual areas in the medio ventral occipital cortex (MVOcC) and medial superior occipital gyrus (msOccG) also exhibited the longest TRW, while in the left hemisphere these regions exhibited shorter processing time scales. See Fig. 4 for a visualization of the TRWs across regions. Figure 5 shows ISC values in selected regions, and Table S2 provides the complete list of ISC values across regions and experimental conditions.

#### Intermediate high-goal TRW

Compared to the intact condition, ISC values were significantly lower in the Primitives and Sub-Goal scramble conditions, but not in the High-Goal scramble condition, in the bilateral middle frontal gyrus (MFG), left SPL and right primary somatosensory cortex (A1/2/3) and MT+ area. Thus, these regions exhibit a relatively long TRW of more than ± 4 s but less than 10 s (see Figs. 4 and 5).

#### Intermediate sub-goal TRW

The lower TRW of the Sub-Goal processing time scale of more than ± 1.5 s but less than ± 4 s includes the bilateral somatosensory A2, the right PMd cortex (A6), and the left ventral premotor (A44d), primary motor (A4ul) and MT+ areas. Thus, these regions show a decrease in ISC only in the Primitive scramble (± 1.5 s) compared to the intact video presentation. The Sub-Goal processing time scale was additionally found in the left lsOccG and early visual cortices (MVOcC, msOccG) while their homotopic regions in the right hemisphere exhibited longer TRWs, as mentioned above (see Figs. 4 and 5).

#### Short primitives TRW

The shortest TRW, of the Primitives time scale, was attributed to regions in which ISC values did *not* decrease in any of the scrambled conditions compared to the intact video presentation (see Fig. 2 for the TRW schematic). Bilaterally, these regions include the anterior inferior parietal lobule (PFop), ventral A44, and inferior occipital gyrus (iOccG). Short TRWs were also found in the right IFJ, dorsal A44 (A44d) and primary motor cortex (A4t). The right IPS (Hip3) also exhibited the lowest TRW time scale while the left IPS exhibited a long processing time scale. The opposite mapping was found for the PFm, such that this region exhibited a short TRW in the left hemisphere and a long TRW in the right hemisphere (see Figs. 4 and 5).

In addition to the aforementioned ROIs, several ROIs with over 30 significant voxels across the experimental conditions showed patterns of ISC levels between conditions that were inconsistent with any of the TRW schemes described in Fig. 2. Thus, these regions could not be assigned with one of the TRWs tested in the current study (see Table S2).

## Discussion

Current theories of action organization postulate hierarchical structuring that is dependent on the temporal dynamics of action goals^14^. Yet thus far a neural mapping of such an action hierarchy was lacking. Therefore, in the current study, we examined whether brain areas in sensory-motor pathways involved in the processing of observed actions, integrate the goal-directed action information over graded timescales. As expected, primary motor areas exhibit low processing temporal scales while regions of the intraparietal sulcus and inferior parietal cortex exhibit the most extended timescales. Interestingly, the primary somatosensory cortex and early visual areas showed relatively long timescales as well. Moreover, the long TRWs in sensory regions were found to be biased to the right hemisphere. Our results conform with a hierarchical topographical mapping of action goals according to increasing processing temporal scales and contribute to prominent arguments of predictive coding mechanisms for inferring other people's goals.

### Areas engaged during action observation exhibit hierarchical temporal scales for processing goal-directed action sequences

In the current study, we examined the neural topography of TRWs among areas responsive to observed everyday actions. The areas found responsive encompass regions of the AON as defined by traditional univariate contrast analysis of BOLD responses^1,32^. In addition to the traditional areas of the AON, the current ISC analysis also revealed voxels in the Precuneus and MFG, and in several early visual cortices. The ISC analysis is indeed complementary to response amplitude methods and can therefore uncover additional phenomena of sensitivity to dynamic stimuli in the brain that are undetectable using univariate measures of response amplitude^16,17^. The voxel-based analysis in the current study revealed the highest ISC values during observation of goal-directed actions in inferior and superior occipital regions, SPL, and somatosensory cortex. Our voxel-based findings are compatible with the study of Thomas and colleagues, who used ISC analysis and showed voxels that are responsive to observed actions in somatosensory, and visual cortices^20^. Beyond the overall high voxel-based ISC levels, our ROI-based ISC analysis revealed hierarchical topographical mapping of goal-directed action sequences across regions in sensory-motor pathways, based on their temporal sensitivity. In both hemispheres, the results show relatively long processing timescales of high-level goal representation in the Precuneus, SPL and MFG, whereas relatively short TRWs, of sub-goal and primitive levels, in the primary motor and ventral premotor cortices.

Our result of long TRW found in the Precuneus is in line with previous studies showing long TRW in this region based on ISC during the processing of visual dynamic stimulus or auditory narratives^17,19^. Moreover, previous reports indicate strong sensitivity of the Precuneus to the end-goal compared with the means of observed actions^33^ and to its contribution to understanding of other people’s intentions and ‘mind-reading’ during first-person perspective taking^34^. Taken together, the findings are compatible with the temporal hierarchy hypothesis^14^, suggesting that intention and goal representation is more stable over time and as such, should require relatively long temporal capacity for information representation.

Although not a core AON region, the MFG was previously reported to encode movement kinematics during goal-directed action observation^4^. While the MFG is frequently associated with cognitive control and action selection^35^ it was also suggested to play a role in maintaining goal-related representations for top-down modulation of sensory activity during working memory^36^. Our results of a long TRW in the MFG are in line with this view, indicating the region’s capacity to integrate action information over a long time window.

In the left hemisphere, the longest TRWs (> ± 10 s) were additionally found in the IPS and PMd cortex while in the right hemisphere, these areas exhibit short TRWs (± 1.5 s < TRW <  ± 4 s, or even TRW <  ± 1.5 s). Interestingly, the opposite trend is evident for sensory regions. In the right hemisphere, longest TRWs were also found in the lateral superior and medio ventral occipital regions and in the PFm, together with a relatively long TRW (± 4 s < TRW <  ± 10 s) in the primary somatosensory cortex, whereas their homotopic regions exhibit relatively short sub-goal processing time scales (± 1.5 s < TRW <  ± 4 s or TRW <  ± 1.5 s) in the left hemisphere.

Within and across hemispheres, the current results indicate that different brain regions process observed actions on (at least) four different temporal scales tested in the current study. Compatible with the framework of action organization suggesting hierarchical structuring that is governed by the temporal dynamics of action goals^14^, the current results indicate a neural mapping of action organization in frontoparietal and visual regions according to the regions’ temporal scales for processing observed actions. Note that several regions did not conform with one of the four TRW categories tested in the current study (see the list of regions in the ‘Unspecified’ TRW section of Table S2). Thus, we cannot determine their position within the temporal hierarchy for processing of goal-directed action sequences.

### Shorter time scales of sequence information processing in the left ventral premotor cortex than dorsal premotor and IPS

Compatible with previous findings, we show that the neural response in parietal and premotor regions is modulated by the temporal coherency of observed actions. Moreover, our results reveal that the different frontoparietal regions exhibit differential levels of temporal sensitivity. Thus, the findings delineate the varying capacities of regions to integrate action information. While the IPL, IPS and ventral premotor (BA44) were previously shown to be sensitive to the temporal structure of observed actions^20,21^, here, we further show that these regions exhibit different temporal capacities to process action goals. Specifically, the left ventral premotor (BA44d) exhibit a Sub-Goal timescale (± 1.5 s < TRW <  ± 4 s) of action processing, mapped to an intermediate level in the action hierarchy, while the right IPL, the left IPS and PMd have the capacity to integrate information over longer time scales, thus mapped to the top of the action hierarchy (TRW >  ± 10 s). The difference we find between the ventral and dorsal regions of the premotor cortex is in line with an anatomical and functional distinction of the two regions, suggesting that they are involved in different aspects of motor behavior in primates. According to this account, the PMv process direct sensorimotor information while the PMd retrieves and integrates, indirect, multiple sets of motor information from sensory signals^37^. A similar segregation between PMv and PMd was also found in an fMRI study using repetition suppression for observed manual object-manipulation in humans^38^. The study showed stronger suppression for hand kinematics in PMv while responses in the PMd were more sensitive to the desired end state of the observed action. The longer TRW we find in the PMd is line with these accounts of higher-level integration of actions in the PMd compared to PMv.

In addition to TRW difference between premotor regions, different TRWs were also found between PMv and parietal regions. Specifically, longer TRWs were found in the left IPS, and right IPL (PFm) compared to the left PMv. While previous results from action execution studies dissociate the level of representation in PMv and parietal regions, pointing to low-level sensory motor representation in the former and a more generalized action goal representation in the latter^39,40^, action observation studies show a less conclusive dissociation. Several action observation studies place the two regions at the same level, showing their involvement in a generalized goal representation independent of low-level sensory motor features^5–7,11^, while others show a different level of representation with the PMv being associated with effector and kinematic dependent action representation, and IPL with more generalized representation^12,13,27,41^. The current results shed new light on these previous findings demonstrating differences in the temporal capacity to integrate observed action information such that PMv process information on a relatively short TRW, compatible with the representation of motor primitives, while the posterior IPL process information on a longer time scale representing the high-level goal information during observation. In light of these reports, our results support the notion that relative to low-level kinematics, observed overarching goals are more stable in time and are thus encoded in areas with a longer temporal capacity to accumulate and integrate action information. In other words, different brain areas have different intrinsic processing characteristics which may determine the neural encoding of observed actions. Accordingly, areas with longer temporal processing capacities constitute the higher levels of the action hierarchy in which low level information is integrated to encode overarching goals, whereas areas with shorter timescales constitute the lower levels and encode only the transient, motor act kinematics^14^.

In the current study, we examined action observation. As similarities were previously found between neural activity during action observation, execution and planning^1,42^, our results regarding temporal timescales might generalize from observation to action planning and execution. Indeed, the long TRWs we find in the IPS and PFm are compatible with patient studies demonstrating a correlation between lesions in these areas and deficits during the planning and execution of goal-directed actions, known as *Ideational apraxia*^43–45^*.* A reduced temporal processing capacity due to such lesions might interfere with the stability of goal representation and be manifested with action planning deficits. It should be noted that in the current study, the PFm and IPS show long temporal processing capacities while other regions of the IPL, such as the more anterior PFop exhibit shorter temporal scales. The parietal lobe is found to be engaged in varying motor as well as other cognitive tasks^46^ and its different sub-regions were shown to play different functional roles both in the current study and in previous findings^25^. Our results, showing a relatively short TRW in the PFop while a longer TRW is found in the PFm are in line with a connectivity-based segregation between the anterior PFop and posterior PFm areas. In a recent thorough investigation of the functional attributes of the parietal cortex using effective connectivity and diffusion tractography, it was shown that compared with the anterior parietal, posterior parietal regions have stronger effective connectivity with visual cortical areas and the hippocampal system, combining visuomotor, visual object, and reward input, while the anterior regions have greater effective connectivity with somatosensory and premotor cortical areas^47^. This is also in line with a recent high-resolution 7T fMRI study showing increased functional connectivity between deep layers of anterior IPL (area PFt) and PMv (BA44) during the observation of intact vs. scrambled action sequences^21^.

### Both primary and high-level areas exhibit long TRWs

Previous studies using ISC analysis of fMRI data parametrically manipulated the temporal coherency of visual^17^, and auditory^19^ input. Their findings revealed that both the visual and auditory systems exhibit hierarchically increasing TRWs that are topographically mapped from primary to high-level areas of the relevant sensory system. Our findings confirm that for action observation as well, brain areas exhibit a hierarchy of TRWs for processing goal-directed action information. As expected, frontoparietal and lateral occipital areas, traditionally considered to encode high-level conceptual information in both action observation^6,7,10,12,13,48^ and execution^39,49–52^, exhibit relatively long TRWs. Interestingly, our results further show that several regions traditionally considered as primary areas exhibit relatively long TRWs as well. Specifically, we find that the primary somatosensory cortex and early visual cortex (EVC) in the right hemisphere exhibit relatively long TRWs of ± 10 s and even longer. Since action observation engages motor, somatosensory and visual pathways^1,2,42,53–55^ the interaction between the different systems may have an effect on the functional characteristics of neural responses within the different areas.

Previous studies have demonstrated the involvement of primary somatosensory cortex (SI) during action observation^1,56,57^. It was suggested that SI, namely BA2, is recruited by the vicarious aspects of the observed action. This may indicate an instantaneous activation, locked to local somatosensory features at the sight of other people’s actions^56^. Interestingly however, it was demonstrated that prior knowledge regarding the goal of an observed action modulates activity within the somatosensory cortex. This was evident even when the object-goal of the action was not visible^57^. This can be taken to suggest that the somatosensory representation of action goals is driven not only by the instantaneously perceived physical features of the movement, but also by the global goal information which is continuous and stable over time. Our findings of long TRWs in right S1 are in line with this notion, indicating the region’s capacity to integrate over goal related action information in a relatively long time window rather than only respond to momentary sensory features of the stimuli.

It was previously suggested that predictive feedback to the occipital cortex from the motor cortex during action execution^58^, and from premotor and parietal areas during observation^20,21^ modulate the neural representation of the visual input such that visual responses represent and send feedforward action prediction errors. Accordingly, observing a scrambled presentation of motor acts hinders goal predictability and therefore elevates prediction errors. This, in turn, might induce divergent neural activity across subjects and, therefore, a reduction in ISC. In addition to feedback connections from the motor cortex, it was shown that primary visual cortex receives strong feedback connections from MT+, LOC and the PFm of the parietal lobe and that high-level information is encoded in EVC during evidence accumulation, prediction and spatial attention^47,59,60^. The involvement of the EVC in such complex perceptual operations was suggested to reflect top-down modulation of activity in primary visual cortex by feedback projections^60^. Moreover, it was suggested that such feedback projections from higher hierarchical levels modulate the temporal dynamics of lower levels^61,62^. Thus, the existence of such top-down modulation by feedback connections may play a role in the reduced ISC levels found in the current study for scrambled actions, and the corresponding long TRWs in primary sensory regions.

### Hemispheric biases

In addition to the differences in processing scales across frontoparietal and occipital regions, our results further revealed differences in TRWs between hemispheres. Specifically, longer TRWs were found in primary somatosensory, MT+, and early visual areas (msOccG and MVOcC) of the right hemisphere. We did not initially expect to find such right lateralization for longer TRWs. Yet, a right hemispheric dominancy for processing static frames depicting coherent vs. non-coherent visual events, was previously shown in areas including the postcentral gyrus as well as middle and posterior superior temporal cortex^63^. Hasson and colleagues directly examined the topographical mapping of TRWs in the visual system using dynamic stimuli, however their results were averaged across hemispheres^17^. Cerliani and colleagues directly examined sensitivity to temporal structures in action observation, however, their analysis was focused on specific regions only in the left hemisphere (PFt, BA44, lateral occipital cortex)^21^.

Taken together, the current results point to a potential advantage of the right hemisphere in the temporal capacity to integrate over observed action sequence information. Hemispheric asymmetry is known for spatial processing of visual input with a right hemispheric specialization for global vs. local processing due to greater sensitivity to low vs. high spatial frequencies in the right compared to the left hemisphere^64^. This is also supported by evidence from patients showing that left hemisphere lesions are associated with deficits in processing local visual features, while patients with right hemisphere lesions are more impaired in global visual processing^65^. A right hemispheric advantage to slow frequencies and hence to global processing in the temporal, as in the spatial aspect of visual inputs, might account for the pattern of results found in the current study. Nevertheless, the question of hemispheric differences in processing scales of observed action information remains for future studies.

Note that due to the approach we used to construct the scrambled videos from the intact video, the four videos differ with respect to low-level visual transients. Thus, the amount of visual transients is greater in the Primitive scramble and is progressively less in the other conditions. It is possible that visual transients induce an evoked response that is common across subjects, thus increasing ISC. In this case, our results would be biased towards higher ISC levels in the scrambled conditions and consequently shift the identification of regions towards having low TRWs. Intriguingly, in early visual cortices the current results show a significant *decrease*, rather than increase, in ISC for the shortest scrambling timescale (Primitives ± 1.5 s) which is compatible with a long TRW and sensitivity to observed action coherency rather than synchronization that is locked to low-level visual transients. In addition, a modulation of ISC due to visual transients would be similar for the two hemispheres. However, the current results show longer TRWs in the right compared to the left hemisphere. Therefore, low-level visual features are less likely to be linked to the increased ISC during the Intact video presentation in the right hemisphere and not in the left. Taken together, the increased number of visual transients in the scrambled conditions seems less likely to explain the pattern of results we obtained in visual cortex. Previous studies, have equated the number of visual transients across conditions by using video recordings from two camera angles and changing the viewpoint angle after each primitive action^20,21^. In the current study we did not implement this approach since we were interested in keeping the intact video, and coherent segments in the scrambled conditions, as natural and similar to action stimuli that we normally perceive in real life.

The current study established the existence of a TRW hierarchy for processing goal-directed actions. While here we show four different time-scales for processing baking-related, goal-directed actions, future investigation are advisable in order to provide a complete characterization of the hierarchy manifestation and its developmental trajectory. Specifically, to understand how the TRWs develop and are shaped by experience, what is the upper limit of processing time-window across regions and whether the hierarchy found here can also be generalized to processing of other types of actions such as intransitive non-goal directed actions or to other contextual domains.

## Conclusion

The current study systematically examined four temporal scales of action goals and demonstrates that, instead of a uniform network, areas in sensory-motor pathways exhibit graded neuro-physiological characteristics of processing capacities during the integration of observed goal-directed action sequences. Our findings delineate a range of timescales across action observation areas showing that the right posterior IPL, and the left IPS and PMd, exhibit the longest TRW, compared to relatively short TRWs in the PMv and primary motor cortices. This is in line with conceptual goal representation previously found in parietal regions, while motor primitives are more frequently associated in the literature with the PMv. Moreover, the results point to a right-hemisphere bias for long TRWs, mainly in the medial occipital cortex. Taken together, the current results shed light on the neural architecture of action organization, support the theory of temporal hierarchical representation of actions (Uithol et al., 2012) and contribute to arguments of predictive coding mechanisms for neural communication between action observation areas. The findings may further provide insight and help delineate dysfunctions in motor disorders such as Ideational Apraxia, in which action goal representation is compromised and can inspire the development of computational models for human action understanding and recognition.

### Supplementary Information

Supplementary Legends.

Supplementary Tables.

Supplementary Video 1.

Supplementary Video 2.

Supplementary Video 3.

Supplementary Video 4.

## Data Availability

According to the conditions of our ethics approval, the data of this study cannot be uploaded to open repository, however, anonymized data can be provided upon request.

## Code availability

Code for data analysis is available at https://github.com/ShaharAb/Temporal_Hierarchy_study.
