# Supplementary material for: Temporal hierarchy of observed goal-directed actions
Source: Sci Rep. 2023 Nov 11;13:19701. doi: 10.1038/s41598-023-46917-z (PMC10640622; doi:10.1038/s41598-023-46917-z)
Supplement: Supplementary file 1 — Supplementary Legends. [file 41598_2023_46917_MOESM1_ESM.pdf]

Supplementary materials for: *Temporal Hierarchy of Observed Goal-Directed Actions*

Shahar Aberbach-Goodman, *Roy Mukamel

Sagol School of Neuroscience and School of Psychological Sciences, Tel Aviv University, Tel-Aviv, Israel, 6997801

*Correspondence to rmukamel@tau.ac.il

Tel: +972-3-640-7246

<u>Video legends:</u>

Video_1 – Intact. Snippet of 30s from the full five minutes video of intact presentation of actions in their chronological order.

Video_2 – High-Goal scramble. Snippet of 30s from the full five minutes video of actions presented in non-chronological order, scrambled at ±10s.

Video_3 – Sub-Goal scramble. Snippet of 30s from the full five minutes video of actions presented in non-chronological order, scrambled at ±4s.

Video_4 – Primitives scramble Snippet of 30s from the full five minutes video of actions presented in non-chronological order, scrambled at ±1.5s.
