# Supplementary material for: Temporal hierarchy of observed goal-directed actions
Source: Sci Rep. 2023 Nov 11;13:19701. doi: 10.1038/s41598-023-46917-z (PMC10640622; doi:10.1038/s41598-023-46917-z)
Supplement: Supplementary file 2 — Supplementary Tables. [file 41598_2023_46917_MOESM2_ESM.pdf]

Supplementary materials for: *Temporal Hierarchy of Observed Goal-Directed Actions*

Shahar Aberbach-Goodman, *Roy Mukamel

Sagol School of Neuroscience and School of Psychological Sciences, Tel Aviv University, Tel-Aviv, Israel, 6997801

***Correspondence to rmukamel@tau.ac.il**

Tel: +972-3-640-7246

Table S1. Stimuli – Goal depiction

List of the observed sub-Goal clips comprising each of the High-Goals, with specification of their durations (in seconds) and their number of primitives.

High-Goals	Sub-Goals	Duration (s)	# Primitives
add egg white to bowl	take bowl	3	2
	take spoon	3	2
	pour egg white	7	4
mix egg white	pass spoon to other hand	2	2
	rotate spoon	4	2
whisk egg white with sugar	pour sugar	3	3
	place back and rotate spoon	4	3
add egg yolks to batter	pour egg yolks	4	2
	place bowl back	4	2
	gentle blend with spoon	3	2
mix egg yolks	rotate spoon	3	2
	jiggle spoon	3	3
	place spoon aside	6	2
add flower	pour flower	3	3
	place cup back	2	2

mix flower	cover flower with batter	4	3
	rotate spoon	2	2
	jiggle spoon	2	2
	place spoon back	3	2
insert batter to mold	grab bowl	1	2
	lift bowl	2	2
	pour mold	6	4
wipe spoon	slide spoon on bowl edge	2	2
	place bowl back	2	2
	jiggle spoon	3	3
	place spoon back	2	2
cut first layer	grab knife	4	3
	slice with knife	8	4
	place knife back	2	2
separate first layer	lift first layer	4	2
	put first layer aside	2	2
cut second layer	slice with knife	5	5
	take out knife	4	4
separate second layer	lift layer	4	3
	put layer aside	2	2
clean crumbs	grab layer	3	2
	sweep crumbs with hand	3	4
add blue color	grab cream	4	2
	open bottle	4	4
	pour blue color	6	4
mix blue color	rotate spoon in cream	5	4
	place bowl back	4	2
add red color	grab cream bowl	3	2
	open red bottle	5	4
	pour red color	5	4
mix red color	rotate spoon in cream	4	3

	place red bowl back	4	4
make red layer	spread red cream	3	3
	place layer on top	9	3
make blue layer	spread blue cream	6	4
	grab layer	6	4
	place layer on top	5	2
decorate first half with waffle	open bag	3	3
	sort waffle on cake	4	3
decorate second half with waffle	twist cake	4	2
	put last waffle	7	3
make glazing circle	grab pastry bag	4	4
	drizzle cream 1	6	3
glaze cake	drizzle cream 2	6	2
	drizzle cream 3	4	2
break cookies	open bag	3	3
	break a cookie	4	3
	break a cookie	5	4
spread cookie crumbs	grab crumbs	3	2
	scatter crumbs	5	2
make first cut in cake	slice with knife	11	3
	pull out knife	4	2
make second cut in cake	slice with knife	8	2
	pull out knife	4	2
take out slice	insert spatula	4	3
	lift cake to plate	9	3
put waffles in plate	place waffle on plate (1)	2	2
	place waffle on plate (2)	4	3

Table S2. ROIs of reliable voxels

Brain regions with at least 30 significantly responsive voxels during at least one of the action observation conditions (see methods). From left to right: TRW label, ROI names according to the Brainnetome atlas nomenclature, number of significant voxels in ROI (and their percentage from total voxels in the atlas ROI), group level mean ISC value and SE (computed for all the ROI's significant voxels) in the four video conditions (P = Primitives, SG = Sub-Goal, HG = High-Goal), used for the subsequent, paired sample T-test analysis.

							MNI Coordinates		
**TRW**	**ROI**	**#V**	**P**	**SG**	**HG**	**Intact**	**X**	**Y**	**Z**
Long	MFG_IFJ_L	494 (81%)	-.02 (.03)	.08 (.03)	.05 (.02)	.17 (.03)	-42	12	38
	MFG_A6vl_L	461 (77%)	0 (.03)	.07 (.03)	.03 (.02)	.17 (.02)	-33	7	55
	PhG_A35/36r_R	38 (22%)	.02 (.01)	.01 (.01)	-.02 (.01)	.07 (.01)	27	-7	-34
	SPL_A7pc_R	477 (92%)	.25 (.03)	.35 (.03)	.4 (.04)	.5 (.04)	23	-47	64
	IPL_A39rd_L	701 (73%)	-.02 (.02)	.07 (.02)	.1 (.02)	.19 (.02)	-38	-64	44
	IPL_A40c_R	971 (89%)	.08 (.02)	.08 (.02)	.07 (.02)	.17 (.03)	55	-49	37
	PCun_A7m_R	498 (95%)	.16 (.02)	.18 (.03)	.17 (.03)	.29 (.03)	5	-64	50
	PCun_A7m_L	422 (99%)	.13 (.02)	.19 (.03)	.18 (.03)	.29 (.03)	-6	-65	51
	MVOcC _rCunG_R	775 (100%)	.32 (.04)	.38 (.04)	.43 (.03)	.5 (.04)	5	-80	10
	LOcC_mOccG_R	787 (95%)	.48 (.03)	.52 (.02)	.53 (.04)	.59 (.03)	30	-89	11
	LOcC _msOccG_R	575 (93%)	.34 (.04)	.4 (.03)	.38 (.03)	.49 (.04)	11	-88	31
	LOcC _lsOccG_R	563 (95%)	.3 (.03)	.37 (.03)	.4 (.04)	.49 (.03)	22	-77	35
	BG_GP_L	123 (39%)	-.01 (.02)	.03 (.02)	.03 (.02)	.1 (.02)	-23	-1	5
	BG_vmPu_L	166 (57%)	0 (.01)	.06 (.02)	.05 (.02)	.14 (.02)	-23	7	-2
	Tha_cTtha_R	102 (48%)	.07 (.02)	.06 (.02)	.05 (.02)	.13 (.02)	12	-20	14

Intermediate HG	MFG_A9/46d_L	424 (42%)	0 (.02)	.05 (.03)	.05 (.02)	.12 (.02)	-30	37	35
	MFG_A46_R	577 (72%)	.04 (.02)	.03 (.02)	.06 (.03)	.15 (.03)	28	56	12
	PhG_TH_R	135 (87%)	.13 (.02)	.13 (.02)	.19 (.02)	.19 (.02)	17	-40	-9
	PhG_TH_L	114 (71%)	.06 (.02)	.08 (.02)	.1 (.02)	.14 (.02)	-19	-37	-11
	SPL_A7pc_L	348 (76%)	.14 (.03)	.19 (.02)	.28 (.03)	.3 (.03)	-23	-43	65
	IPL_A39c_R	933 (90%)	.37 (.03)	.42 (.03)	.46 (.04)	.52 (.04)	34	-80	29
	PCun_A5m_R	561 (97%)	.13 (.02)	.15 (.02)	.24 (.03)	.28 (.03)	8	-47	57
	PCun_dmPOS_R	875 (100%)	.19 (.02)	.2 (.03)	.25 (.02)	.31 (.03)	12	-67	25
	PCun_dmPOS_L	920 (88%)	.14 (.02)	.14 (.02)	.19 (.02)	.25 (.03)	-16	-65	26
	PoG_A1/2/3tru_R	407 (60%)	.05 (.02)	.08 (.02)	.12 (.02)	.15 (.03)	24	-35	66
	MVOcC _cLinG_R	486 (100%)	.5 (.04)	.49 (.04)	.54 (.05)	.57 (.04)	10	-83	-11
	MVOcC _rLinG_R	759 (100%)	.34 (.04)	.36 (.04)	.43 (.03)	.47 (.04)	16	-61	-6
	MVOcC _vmPOS_R	999 (100%)	.25 (.03)	.3 (.04)	.35 (.02)	.42 (.04)	13	-68	12
	MVOcC _vmPOS_L	1000 (95%)	.19 (.02)	.25 (.03)	.27 (.02)	.35 (.03)	-14	-64	13
	LOcC _V5/MT+_R	733 (97%)	.42 (.03)	.43 (.03)	.48 (.03)	.52 (.03)	46	-74	3
	Tha_PPtha_R	150 (61%)	.08 (.02)	.07 (.02)	.08 (.02)	.14 (.02)	17	-25	6
Intermediate SG	SFG_A8dl_L	370 (51%)	0 (.02)	.12 (.03)	.05 (.02)	.08 (.02)	-22	26	50
	SFG_A6dl_R	548 (84%)	.12 (.03)	.16 (.02)	.19 (.03)	.21 (.04)	19	-1	64
	SFG_A6dl_L	508 (75%)	.08 (.03)	.11 (.02)	.11 (.02)	.17 (.02)	-21	4	63
	SFG_A9m_L	553 (62%)	.02 (.02)	.1 (.03)	.06 (.02)	.14 (.03)	-6	35	35
	MFG_A9/46v_L	420 (45%)	-.01 (.02)	.08 (.02)	.04 (.03)	.13 (.03)	-41	41	14
	MFG_A8vl_R	651 (65%)	.06 (.02)	.08 (.02)	.07 (.02)	.12 (.02)	33	22	46
	MFG_A8vl_L	353 (43%)	-.06 (.03)	.06 (.03)	.07 (.02)	.14 (.02)	-43	26	36
	MFG_A6vl_R	619 (97%)	.16 (.02)	.15 (.03)	.19 (.03)	.23 (.03)	32	4	55
	MFG_A10l_R	349 (43%)	.04 (.02)	.07 (.02)	.06 (.02)	.13 (.03)	26	60	-3
	MFG_A10l_L	291 (28%)	-.02 (.03)	.08 (.02)	.08 (.02)	.14 (.03)	-25	61	1
	IFG_A44d_L	270 (79%)	0 (.03)	.09 (.02)	.08 (.03)	.14 (.03)	-45	17	26
	IFG_IFS_L	259 (80%)	-.02 (.02)	.13 (.02)	.06 (.03)	.1 (.03)	-47	34	14
	IFG_A45c_L	148 (40%)	-.06 (.02)	.1 (.03)	.08 (.02)	.05 (.03)	-54	25	16
	IFG_A44op_L	199 (36%)	.03 (.02)	.06 (.02)	.07 (.02)	.12 (.02)	-42	19	4
	PrG_A4hf_L	306 (59%)	.2 (.03)	.29 (.03)	.26 (.04)	.28 (.04)	-56	-1	34
	PrG_A6cdl_R	707 (99%)	.26 (.03)	.34 (.03)	.38 (.03)	.41 (.04)	32	-9	58
	PrG_A6cdl_L	846 (96%)	.18 (.03)	.29 (.02)	.26 (.04)	.3 (.03)	-32	-7	57
	PrG_A4ul_L	534 (85%)	.04 (.02)	.09 (.02)	.14 (.03)	.13 (.03)	-35	-19	59
	PrG_A6cvl_L	637 (94%)	.09 (.03)	.21 (.03)	.2 (.03)	.18 (.03)	-51	7	30

	ITG_A37elv_R	332 (100%)	.27 (.03)	.29 (.02)	.32 (.04)	.34 (.03)	50	-57	-14
	ITG_A37elv_L	181 (84%)	.08 (.02)	.15 (.03)	.14 (.03)	.15 (.03)	-53	-54	-18
	ITG_A37vl_R	405 (98%)	.29 (.03)	.3 (.02)	.37 (.04)	.38 (.04)	55	-60	-6
	ITG_A37vl_L	294 (83%)	.07 (.03)	.12 (.03)	.16 (.04)	.19 (.03)	-53	-57	-8
	FuG_A20rv_R	374 (37%)	.21 (.03)	.25 (.02)	.3 (.02)	.31 (.03)	32	-30	-22
	FuG_A20rv_L	291 (27%)	.07 (.02)	.14 (.02)	.15 (.02)	.19 (.02)	-34	-29	-23
	FuG_A37mv_R	893 (100%)	.5 (.03)	.52 (.03)	.54 (.04)	.56 (.03)	31	-65	-14
	FuG_A37mv_L	806 (100%)	.48 (.03)	.52 (.03)	.48 (.04)	.53 (.03)	-31	-61	-14
	FuG_A37lv_L	825 (92%)	.24 (.03)	.3 (.03)	.28 (.04)	.33 (.03)	-43	-50	-18
	PhG_TL_R	149 (89%)	.12 (.02)	.17 (.02)	.2 (.02)	.24 (.02)	28	-32	-17
	PhG_TL_L	87 (64%)	.03 (.01)	.1 (.02)	.07 (.02)	.12 (.01)	-29	-31	-18
	pSTS_rpSTS_R	267 (86%)	.06 (.02)	.11 (.03)	.13 (.02)	.17 (.02)	54	-41	4
	pSTS_cpSTS_R	349 (96%)	.1 (.02)	.2 (.03)	.2 (.03)	.21 (.03)	53	-50	11
	SPL_A7r_R	335 (92%)	.29 (.03)	.4 (.03)	.43 (.04)	.48 (.04)	17	-60	62
	SPL_A7r_L	388 (72%)	.36 (.04)	.46 (.03)	.43 (.04)	.5 (.03)	-20	-56	64
	SPL_A7c_R	461 (97%)	.25 (.03)	.29 (.03)	.32 (.04)	.36 (.04)	16	-71	52
	SPL_A7c_L	476 (97%)	.28 (.03)	.37 (.03)	.36 (.03)	.4 (.03)	-18	-69	54
	SPL_A5l_R	475 (99%)	.27 (.03)	.29 (.03)	.35 (.04)	.37 (.05)	33	-47	50
	SPL_A5l_L	342 (93%)	.28 (.04)	.4 (.03)	.4 (.04)	.42 (.03)	-35	-42	53
	SPL_A7ip_R	401 (99%)	.32 (.03)	.36 (.03)	.4 (.04)	.44 (.04)	27	-59	54
	SPL_A7ip_L	443 (93%)	.27 (.03)	.36 (.03)	.36 (.04)	.37 (.03)	-31	-54	53
	IPL_A39c_L	1090 (93%)	.16 (.03)	.24 (.03)	.27 (.03)	.28 (.03)	-45	-72	20
	IPL_A40rd_L	945 (93%)	.26 (.04)	.34 (.03)	.37 (.04)	.34 (.03)	-46	-35	45
	IPL_A39rv_L	923 (69%)	.05 (.03)	.13 (.03)	.08 (.02)	.12 (.03)	-53	-53	22
	PCun_A5m_L	599 (89%)	.12 (.02)	.15 (.02)	.21 (.03)	.23 (.03)	-7	-47	57
	PoG_A1/2/3tonIa_R	552 (84%)	.15 (.03)	.19 (.03)	.2 (.04)	.24 (.04)	57	-14	16
	PoG_A2_R	720 (98%)	.34 (.04)	.42 (.04)	.44 (.04)	.47 (.05)	46	-30	50
	PoG_A2_L	679 (89%)	.1 (.03)	.19 (.03)	.21 (.04)	.21 (.03)	-48	-24	48
	CG_A23v_R	341 (95%)	.13 (.02)	.17 (.02)	.17 (.02)	.21 (.02)	8	-47	9
	CG_A23v_L	217 (69%)	.1 (.01)	.11 (.02)	.12 (.02)	.17 (.02)	-8	-44	10
	CG_A23c_R	539 (88%)	.1 (.02)	.1 (.02)	.15 (.02)	.15 (.02)	7	-23	41
	MVOcC _rCunG_L	825 (100%)	.3 (.03)	.42 (.03)	.41 (.03)	.47 (.04)	-7	-76	11
	MVOcC _cCunG_L	636 (100%)	.46 (.04)	.58 (.03)	.52 (.03)	.56 (.04)	-8	-90	12
	MVOcC _rLinG_L	851 (99%)	.38 (.04)	.46 (.03)	.42 (.04)	.48 (.04)	-18	-60	-7
	LOcC_mOccG_L	841 (100%)	.45 (.03)	.49 (.03)	.46 (.04)	.52 (.03)	-34	-86	11
	LOcC _V5/MT+_L	801 (99%)	.37 (.04)	.41 (.03)	.43 (.04)	.43 (.03)	-48	-70	-1
	LOcC _OPC_R	731 (75%)	.49 (.03)	.51 (.02)	.49 (.03)	.54 (.03)	19	-99	4

	LOcC _msOccG_L	636 (99%)	.39 (.03)	.45 (.02)	.42 (.04)	.49 (.03)	-16	-85	34
	LOcC _lsOccG_L	771 (99%)	.21 (.03)	.28 (.03)	.29 (.03)	.34 (.03)	-29	-74	36
	Hipp_rHipp_L	92 (19%)	0 (.01)	.1 (.02)	.06 (.02)	.07 (.02)	-23	-18	-16
	Hipp_cHipp_L	198 (32%)	.05 (.02)	.08 (.02)	.09 (.02)	.1 (.02)	-25	-32	-6
	BG_vCa_R	222 (52%)	.03 (.02)	.08 (.02)	.05 (.02)	.1 (.02)	12	14	0
	BG_vCa_L	173 (55%)	.03 (.01)	.11 (.01)	.06 (.02)	.1 (.02)	-15	13	-2
	BG_NAC_R	149 (43%)	.02 (.02)	.09 (.02)	.04 (.01)	.08 (.01)	18	4	-9
	BG_NAC_L	136 (32%)	.03 (.01)	.1 (.02)	.04 (.01)	.09 (.02)	-18	4	-8
	BG_dlPu_L	247 (45%)	-.02 (.02)	.06 (.02)	.05 (.02)	.09 (.02)	-30	-4	1
	Tha_mPFtha_L	79 (43%)	.03 (.02)	.1 (.02)	.04 (.01)	.09 (.02)	-8	-9	7
	Tha_rTtha_L	85 (40%)	.05 (.01)	.09 (.02)	.07 (.01)	.1 (.02)	-2	-13	5
	Tha_Otha_L	110 (66%)	.05 (.01)	.1 (.01)	.09 (.01)	.11 (.02)	-16	-27	7
	Tha_cTtha_L	101 (64%)	.05 (.02)	.09 (.02)	.07 (.01)	.11 (.02)	-8	-11	13
**Short**	SFG_A8m_R	634 (82%)	.11 (.02)	.1 (.02)	.09 (.02)	.16 (.03)	4	16	53
	SFG_A8dl_R	299 (33%)	.04 (.02)	.07 (.02)	.03 (.01)	.07 (.02)	19	21	54
	SFG_A9l_R	220 (29%)	.01 (.02)	.1 (.02)	.01 (.02)	.05 (.02)	11	45	44
	SFG_A9l_L	535 (58%)	0 (.03)	.15 (.03)	.04 (.02)	.02 (.02)	-13	49	40
	SFG_A6m_R	649 (86%)	.12 (.03)	.12 (.03)	.16 (.02)	.14 (.02)	6	-5	57
	SFG_A6m_L	534 (72%)	.11 (.03)	.13 (.03)	.13 (.02)	.11 (.02)	-7	-4	60
	SFG_A10m_R	437 (41%)	.03 (.02)	.14 (.02)	.07 (.02)	.06 (.02)	7	51	14
	SFG_A10m_L	330 (36%)	.04 (.02)	.15 (.03)	.06 (.03)	.06 (.03)	-7	57	12
	MFG_IFJ_R	732 (93%)	.13 (.02)	.16 (.02)	.13 (.02)	.19 (.03)	42	13	36
	MFG_A9/46v_R	695 (68%)	.13 (.02)	.07 (.02)	.06 (.02)	.13 (.02)	41	40	16
	IFG_A44d_R	315 (97%)	.17 (.02)	.16 (.02)	.15 (.03)	.16 (.03)	46	13	24
	IFG_A45c_R	220 (70%)	.12 (.02)	.09 (.03)	.06 (.02)	.13 (.02)	53	23	12
	IFG_A45r_L	186 (45%)	.01 (.02)	.12 (.03)	.04 (.02)	0 (.02)	-50	38	0
	IFG_A44op_R	297 (65%)	.11 (.02)	.07 (.02)	.06 (.02)	.13 (.03)	39	23	4
	IFG_A44v_R	207 (73%)	.15 (.02)	.04 (.02)	.06 (.03)	.04 (.02)	52	13	7
	IFG_A44v_L	116 (42%)	.03 (.02)	.04 (.02)	.08 (.03)	.03 (.02)	-53	13	12
	OrG_A14m_R	216 (42%)	.09 (.02)	.15 (.03)	.05 (.02)	.05 (.02)	6	52	-6
	OrG_A14m_L	394 (63%)	.1 (.02)	.17 (.03)	.03 (.02)	.05 (.03)	-6	49	-6
	OrG_A12/47o_L	181 (38%)	.03 (.03)	.11 (.03)	-.03 (.02)	.03 (.03)	-40	37	-15
	OrG_A11l_L	274 (23%)	.03 (.02)	.11 (.02)	.03 (.02)	.05 (.02)	-25	32	-17
	OrG_A11m_L	101 (14%)	.02 (.01)	.11 (.03)	0 (.02)	.06 (.02)	-6	52	-18
	OrG_A13_R	191 (21%)	.05 (.02)	.1 (.02)	.02 (.02)	.04 (.02)	15	15	-18
	OrG_A13_L	178 (22%)	.02 (.02)	.08 (.03)	.07 (.02)	-.01 (.02)	-8	25	-19

	OrG_A12/47l_L	218 (43%)	.02 (.02)	.13 (.03)	-.02 (.01)	.02 (.03)	-42	33	-9
	PrG_A4hf_R	606 (68%)	.08 (.02)	.07 (.02)	.06 (.02)	.09 (.02)	48	-8	42
	PrG_A4t_R	202 (69%)	.08 (.02)	.1 (.02)	.1 (.02)	.14 (.02)	15	-19	73
	PrG_A4tl_R	321 (75%)	.08 (.02)	.07 (.02)	.07 (.02)	.04 (.01)	51	0	8
	PrG_A4tl_L	330 (77%)	.09 (.02)	.11 (.02)	.16 (.02)	.04 (.02)	-54	3	8
	PrG_A6cvl_R	690 (92%)	.22 (.02)	.18 (.03)	.2 (.03)	.21 (.03)	50	5	30
	PCL_A1/2/3ll_R	296 (87%)	.08 (.02)	.05 (.02)	.11 (.03)	.13 (.03)	7	-38	58
	PCL_A1/2/3ll_L	275 (59%)	.05 (.02)	.04 (.02)	.14 (.03)	.08 (.02)	-11	-34	52
	PCL_A4ll_R	279 (57%)	.09 (.02)	.07 (.02)	.06 (.02)	.09 (.02)	4	-23	59
	PCL_A4ll_L	410 (66%)	.08 (.02)	.06 (.02)	.09 (.02)	.1 (.02)	-4	-20	61
	STG_A38m_R	163 (23%)	.04 (.02)	.06 (.02)	.07 (.02)	.06 (.02)	34	16	-31
	STG_A41/42_R	291 (60%)	.09 (.02)	.1 (.02)	.13 (.02)	.14 (.03)	56	-33	14
	STG_A41/42_L	324 (88%)	.08 (.02)	.12 (.02)	.15 (.03)	.08 (.02)	-53	-24	11
	STG_TE1.0 TE1.2_R	365 (45%)	.07 (.02)	.08 (.02)	.06 (.02)	.08 (.02)	50	-11	2
	STG_TE1.0 TE1.2_L	528 (73%)	.12 (.02)	.11 (.02)	.15 (.03)	.08 (.03)	-51	-4	0
	STG_A22c_R	402 (68%)	.12 (.03)	.13 (.03)	.12 (.02)	.19 (.03)	64	-37	10
	STG_A22c_L	267 (48%)	.12 (.02)	.12 (.02)	.11 (.03)	.04 (.02)	-65	-23	9
	STG_A38l_R	223 (44%)	.09 (.02)	.07 (.02)	.05 (.01)	.05 (.02)	46	13	-19
	STG_A38l_L	217 (33%)	.08 (.02)	.08 (.03)	.05 (.02)	.05 (.02)	-47	11	-18
	STG_A22r_R	324 (50%)	.08 (.03)	.09 (.02)	.1 (.02)	.13 (.02)	54	-3	-12
	STG_A22r_L	195 (51%)	.09 (.02)	.1 (.03)	.04 (.02)	.08 (.02)	-56	-10	-6
	MTG_A21c_R	227 (41%)	.08 (.02)	.06 (.02)	.08 (.02)	.09 (.02)	65	-32	-10
	MTG_A21r_R	191 (25%)	.06 (.02)	.06 (.03)	.02 (.02)	.03 (.02)	54	3	-30
	MTG_A21r_L	359 (37%)	.04 (.02)	.11 (.03)	.01 (.02)	.05 (.02)	-51	4	-31
	MTG_A37dl_R	573 (92%)	.25 (.03)	.26 (.03)	.31 (.03)	.3 (.03)	59	-57	4
	MTG_A37dl_L	521 (72%)	.06 (.02)	.11 (.03)	.17 (.04)	.13 (.03)	-58	-55	4
	MTG_aSTS_R	433 (51%)	.08 (.02)	.09 (.02)	.11 (.02)	.13 (.03)	57	-19	-10
	MTG_aSTS_L	618 (52%)	.06 (.02)	.14 (.04)	.01 (.02)	.06 (.02)	-58	-14	-10
	ITG_A20iv_R	166 (55%)	.13 (.02)	.16 (.02)	.15 (.03)	.18 (.02)	46	-31	-24
	ITG_A20r_L	30 (7%)	0 (.02)	.06 (.02)	.01 (.01)	0 (.02)	-39	4	-41
	ITG_A20il_R	141 (30%)	.1 (.03)	.06 (.02)	.02 (.02)	.04 (.03)	57	-13	-31
	ITG_A20il_L	64 (14%)	-.02 (.02)	.1 (.02)	-.01 (.02)	0 (.02)	-56	-11	-31
	ITG_A20cl_R	307 (62%)	.16 (.03)	.09 (.02)	.12 (.03)	.13 (.02)	58	-44	-15
	ITG_A20cl_L	95 (23%)	.02 (.01)	.09 (.02)	.04 (.02)	.05 (.02)	-60	-44	-16
	ITG_A20cv_R	192 (33%)	.12 (.03)	.11 (.02)	.1 (.02)	.1 (.02)	54	-34	-26
	ITG_A20cv_L	66 (11%)	.05 (.01)	.09 (.01)	.07 (.01)	.08 (.02)	-52	-38	-24
	FuG_A37lv_R	948 (97%)	.31 (.03)	.36 (.03)	.35 (.04)	.37 (.03)	42	-50	-18

	PhG_A35/36r_L	51 (35%)	0 (.02)	.06 (.01)	.01 (.01)	.04 (.01)	-28	-10	-34
	PhG_A35/36c_R	43 (27%)	.05 (.02)	.05 (.02)	.07 (.01)	.1 (.02)	24	-29	-22
	PhG_A35/36c_L	68 (43%)	.01 (.02)	.07 (.01)	.04 (.01)	.04 (.01)	-26	-26	-26
	PhG_A28/34_R	32 (17%)	.01 (.01)	.02 (.01)	.03 (.01)	.04 (.01)	18	-8	-31
	pSTS_rpSTS_L	159 (47%)	.02 (.02)	.06 (.03)	.1 (.03)	.04 (.02)	-54	-38	5
	pSTS_cpSTS_L	268 (89%)	.09 (.02)	.09 (.02)	.15 (.03)	.1 (.03)	-57	-40	12
	IPL_A39rd_R	793 (98%)	.17 (.02)	.17 (.02)	.19 (.03)	.23 (.03)	38	-61	46
	IPL_A40rd_R	997 (97%)	.22 (.03)	.24 (.03)	.27 (.04)	.24 (.04)	51	-33	41
	IPL_A40c_L	850 (73%)	.06 (.02)	.06 (.02)	.05 (.03)	.09 (.02)	-56	-44	38
	IPL_A39rv_R	1422 (91%)	.19 (.02)	.19 (.03)	.2 (.02)	.25 (.03)	46	-65	26
	IPL_A40rv_R	910 (85%)	.12 (.02)	.12 (.02)	.17 (.03)	.15 (.03)	55	-31	23
	IPL_A40rv_L	1138 (89%)	.2 (.04)	.28 (.03)	.33 (.04)	.24 (.04)	-56	-26	26
	PCun_A31_R	648 (83%)	.07 (.02)	.13 (.02)	.13 (.02)	.14 (.03)	7	-54	35
	PCun_A31_L	738 (79%)	.08 (.02)	.13 (.03)	.1 (.02)	.1 (.03)	-6	-54	35
	PoG_A1/2/3ulhf_L	604 (66%)	.07 (.03)	.07 (.02)	.14 (.03)	.08 (.03)	-47	-17	49
	PoG_A1/2/3tonIa_L	436 (71%)	.08 (.03)	.15 (.02)	.18 (.03)	.13 (.03)	-56	-10	15
	INS_G_R	116 (36%)	.03 (.02)	.07 (.01)	.06 (.01)	.08 (.02)	38	-19	9
	INS_G_L	148 (54%)	.03 (.01)	.07 (.02)	.08 (.02)	.04 (.02)	-37	-18	8
	INS_vIa_R	164 (69%)	.1 (.02)	.09 (.01)	.06 (.01)	.06 (.02)	33	15	-13
	INS_vIa_L	129 (62%)	.04 (.02)	.07 (.02)	.06 (.02)	.08 (.02)	-33	14	-12
	INS_dIa_R	144 (58%)	.09 (.02)	.09 (.01)	.08 (.02)	.13 (.02)	35	19	1
	INS_dIa_L	141 (63%)	.05 (.02)	.05 (.02)	.05 (.02)	.11 (.02)	-36	19	1
	INS_vId/vIg_R	140 (52%)	.07 (.02)	.07 (.01)	.02 (.02)	.07 (.02)	38	-2	-10
	INS_vId/vIg_L	110 (38%)	.05 (.01)	.05 (.02)	.07 (.02)	.05 (.02)	-39	-2	-9
	INS_dIg_R	208 (73%)	.07 (.02)	.07 (.02)	.07 (.02)	.08 (.02)	39	-8	8
	INS_dIg_L	182 (67%)	.08 (.02)	.14 (.02)	.13 (.02)	.1 (.02)	-40	-6	6
	INS_dId_R	241 (59%)	.06 (.01)	.08 (.01)	.02 (.01)	.06 (.02)	39	5	5
	INS_dId_L	256 (83%)	.06 (.02)	.08 (.02)	.1 (.02)	.05 (.02)	-38	5	5
	CG_A23d_R	406 (84%)	.11 (.02)	.1 (.02)	.12 (.02)	.13 (.02)	4	-38	32
	CG_A23d_L	315 (77%)	.1 (.01)	.08 (.02)	.09 (.02)	.13 (.02)	-4	-36	32
	CG_A24rv_R	168 (79%)	.12 (.02)	.14 (.02)	.09 (.02)	.12 (.02)	3	7	26
	CG_A24rv_L	206 (59%)	.09 (.02)	.13 (.02)	.08 (.01)	.09 (.02)	-4	21	12
	CG_A32p_R	448 (95%)	.11 (.02)	.12 (.02)	.09 (.02)	.15 (.02)	5	34	20
	CG_A24cd_R	343 (94%)	.1 (.02)	.12 (.02)	.11 (.02)	.1 (.02)	4	6	38
	CG_A24cd_L	248 (97%)	.11 (.02)	.12 (.02)	.11 (.02)	.09 (.02)	-4	6	38
	CG_A23c_L	461 (84%)	.08 (.02)	.08 (.02)	.13 (.02)	.12 (.02)	-6	-21	41
	CG_A32sg_R	435 (67%)	.08 (.02)	.13 (.02)	.08 (.02)	.09 (.02)	4	41	-1

	CG_A32sg_L	299 (72%)	.07 (.02)	.12 (.02)	.06 (.02)	.12 (.02)	-6	41	8
	MVOcC _cLinG_L	596 (100%)	.59 (.04)	.64 (.03)	.62 (.04)	.63 (.04)	-10	-86	-9
	LOcC _OPC_L	985 (99%)	.52 (.03)	.56 (.02)	.5 (.03)	.55 (.03)	-22	-97	4
	LOcC_iOccG_R	961 (90%)	.41 (.03)	.45 (.03)	.44 (.03)	.45 (.04)	31	-87	-12
	LOcC_iOccG_L	949 (99%)	.47 (.03)	.49 (.03)	.45 (.03)	.5 (.03)	-32	-85	-12
	Amyg_mAmyg_R	45 (26%)	.01 (.02)	.04 (.02)	.04 (.02)	.05 (.02)	20	-1	-19
	Amyg_mAmyg_L	70 (32%)	.02 (.02)	.07 (.02)	.05 (.02)	.04 (.01)	-20	-2	-18
	Amyg_lAmyg_R	31 (36%)	.05 (.02)	.02 (.01)	.06 (.01)	.03 (.01)	26	-5	-16
	Amyg_lAmyg_L	38 (28%)	.02 (.01)	.05 (.02)	.06 (.02)	.04 (.01)	-26	-4	-16
	Hipp_rHipp_R	185 (32%)	.07 (.02)	.09 (.02)	.09 (.01)	.07 (.01)	22	-17	-17
	Hipp_cHipp_R	212 (36%)	.06 (.02)	.09 (.02)	.1 (.02)	.11 (.02)	27	-32	-9
	BG_dCa_R	250 (48%)	.05 (.02)	.07 (.02)	.04 (.02)	.12 (.02)	14	0	17
	BG_dCa_L	256 (38%)	.05 (.02)	.11 (.02)	.08 (.02)	.1 (.02)	-12	7	12
	Tha_mPFtha_R	149 (75%)	.09 (.02)	.09 (.02)	.05 (.01)	.06 (.01)	6	-13	4
	Tha_PPtha_L	127 (60%)	.07 (.02)	.13 (.01)	.12 (.01)	.12 (.02)	-15	-25	5
	Tha_lPFtha_R	116 (32%)	.07 (.01)	.05 (.02)	.04 (.01)	.05 (.01)	10	-15	1
	Tha_lPFtha_L	81 (29%)	.03 (.02)	.08 (.01)	.07 (.01)	.07 (.02)	-13	-19	7
**Unspecified**	SFG_A8m_L	510 (59%)	.05 (.02)	.06 (.03)	.07 (.02)	.14 (.03)	-6	17	52
	SFG_A9m_R	572 (81%)	.06 (.02)	.12 (.02)	.07 (.02)	.16 (.02)	5	34	39
	MFG_A9/46d_R	626 (58%)	.08 (.02)	.09 (.02)	.05 (.02)	.13 (.02)	28	43	31
	MFG_A46_L	722 (70%)	-.03 (.02)	.11 (.03)	.06 (.02)	.16 (.03)	-27	54	15
	IFG_IFS_R	281 (73%)	.14 (.02)	.12 (.02)	.07 (.02)	.16 (.02)	46	31	15
	IFG_A45r_R	220 (58%)	.1 (.02)	.1 (.03)	.02 (.02)	.11 (.03)	49	35	-3
	OrG_A12/47o_R	245 (47%)	.07 (.02)	.08 (.02)	0 (.02)	.09 (.02)	35	33	-15
	OrG_A11l_R	444 (48%)	.07 (.02)	.06 (.02)	.01 (.02)	.12 (.02)	23	37	-16
	OrG_A11m_R	40 (6%)	-.02 (.02)	.07 (.02)	-.03 (.01)	.12 (.02)	12	48	-19
	OrG_A12/47l_R	290 (50%)	.11 (.02)	.11 (.02)	.02 (.02)	.11 (.03)	40	33	-9
	PrG_A4ul_R	486 (79%)	.07 (.02)	.06 (.02)	.09 (.01)	.13 (.03)	28	-24	63
	PrG_A4t_L	341 (78%)	.11 (.02)	.06 (.02)	.11 (.02)	.14 (.02)	-16	-21	71
	STG_A38m_L	62 (9%)	-.02 (.01)	.06 (.02)	0 (.02)	.07 (.01)	-34	16	-29
	MTG_A21c_L	128 (18%)	-.03 (.02)	.09 (.03)	-.05 (.02)	.06 (.02)	-64	-31	-11
	ITG_A20r_R	113 (24%)	.02 (.01)	.02 (.02)	.05 (.01)	.07 (.02)	43	1	-40
	PoG_A1/2/3ulhf_R	705 (77%)	.1 (.03)	.08 (.03)	.12 (.03)	.16 (.04)	49	-18	46
	PoG_A1/2/3tru_L	331 (62%)	.07 (.03)	.07 (.02)	.14 (.02)	.14 (.02)	-23	-32	66
	CG_A32p_L	368 (98%)	.11 (.02)	.1 (.02)	.1 (.02)	.17 (.02)	-5	27	28
	MVOcC _cCunG_R	534 (88%)	.42 (.03)	.41 (.03)	.42 (.04)	.49 (.03)	6	-94	1

	BG_GP_R	101 (31%)	.02 (.01)	.05 (.01)	.03 (.02)	.09 (.02)	23	1	6
	BG_vmPu_R	220 (66%)	.05 (.01)	.08 (.02)	.04 (.02)	.13 (.02)	24	7	-4
	BG_dlPu_R	247 (40%)	0 (.02)	.07 (.02)	.02 (.02)	.1 (.02)	29	-4	-1
	Tha_Stha_R	44 (32%)	.03 (.01)	.01 (.01)	-.01 (.01)	.05 (.01)	18	-23	3
	Tha_rTtha_R	104 (51%)	.09 (.02)	.06 (.01)	.06 (.02)	.12 (.02)	6	-10	7
	Tha_Otha_R	147 (60%)	.13 (.02)	.11 (.02)	.1 (.01)	.15 (.01)	17	-30	2
